# Organophosphate-related ototoxicity: Description of the vestibulocochlear system ultrastructural aspects of guinea pigs

**DOI:** 10.1590/S1808-86942010000200015

**Published:** 2015-10-19

**Authors:** Daiane Körbes, Aron Ferreira da Silveira, Miguel Ângelo Hyppolito, Gisiane Munaro

**Affiliations:** Speech therapist, master's degree student in the Human Communication Disorders graduate program, Santa Maria Federal University, Santa Maria, RS, Brazil; Full professor, head of the Morphology Department, Santa Maria Federal University, Santa Maria, RS, Brazil; Adjunct professor, faculty member of the Ophthalmology, Otorhinolaryngology and Head & Neck Surgery Department, Ribeirao Preto Medical School, Sao Paulo University, SP, Brazil; Speech therapist, master's degree student in the Human Communication Disorders graduate program, Santa Maria Federal University, Santa Maria, RS, Brazil

**Keywords:** microscopy, labyrinth, insecticides, vestibule, organophosphates

## Abstract

Organophosphate toxic agents are used in agriculture and are currently part of the group of toxic agents which can lead to hearing loss, in which we have solvents, metals and asphyxiation agents.

**Aim:**

to analyze the acute ototoxic action of a group of organophosphate agents in the vestibulocochlear system. This is a prospective experimental study.

**Materials and Methods:**

we used male albino guinea pigs, broken down into three groups, to which we provided distilled water (group 1 - control), agrotoxic agent – 0.3mg/Kg/day (group 2), agrotoxic – 3 mg/Kg/day (group 3), during 7 seven consecutive days. The most used agrotoxic agent was Tamaron BR (metamidophos). The anatomical evaluation of the cochlea, saccule and utricle was carried out by means of electronic scanning microscopy after the use of the agrotoxic agent.

**Results:**

the guinea pigs submitted to the organophosphate presented cochlear morphological alterations with lesions on the three turns analyzed, as well as cilia alterations in the saccule and utricle, intensified according to the agent dosage.

**Conclusion:**

the morphological alterations seen in the hair cells exposed to daily doses of organophosphate promote evidences of an acute deleterious effect of agrotoxic agents on the vestibulocochlear system.

## INTRODUCTION

Farmers are exposed to several health hazards such as noise, vibrations, and specific chemical products (agrochemicals). These agents may act simultaneously, which may cause hearing loss. In most cases, farmers in contact with several types of agrochemicals enjoy no periodic monitoring of their auditory health. Because of the effect of these factors on the auditory system, it is possible that farmers are hearing loss candidates.^1^

Recently, organophosphorus agrochemicals were included in the high-priority group for studying ototoxicity due to occupational exposure; this group already included industrial solvents, heavy metals, and various other chemical compounds that commonly as asphyxiating, such as carbon monoxide and hydrogen cyanide.^2^, ^3^, ^4^

Intoxication may occur by inhalation, ingestion or skin contact.^5^^,^^6^ Several clinical manifestations may be observed as a result of toxic effects of agrochemicals on human health, such as nausea, dizziness, tinnitus, weakness, loss of appetite, nervousness, headache, allergies, kidney and liver injury, and cancer.^7^, ^8^, ^9^, ^10^, ^11^ Because intoxication symptoms are non-specific and has multiple etiologies, it is often hard to establish a cause-effect between handling an organophosphorus substance and the manifestations.

Ototoxic substances acting on the vestibulocochlear system may damage the outer hair cells (OHC), the 8th cranial nerve, the vestibular system and the central nervous system (CNS). Neurotoxic chemical substances found in workplaces may affect hearing, balance, the brainstem, and central auditory pathways.^12^ There is evidence that hearing loss may be an early manifestation of organophosphorus intoxication.^1^

There are many scientifically known changes due to organophosphorus intoxication in human beings, which may affect every organ or system in the body. Although the literature is clear on the association between exposure to agrochemicals and hearing loss, there are few studies that have assessed the histological changes specifically in the vestibulocochlear system.

Studies in guinea-pigs are important, given the anatomic and physiological similarities between their peripheral auditory systems and that of human beings, and findings may be correlated with the human population to investigate and alert about possible auditory involvement due to contact with organophosphorus substances.

Therefore, we sought to study the acute ototoxic effect of an organophosphorus agrochemical on the vestibulocochlear system of guinea-pigs.

## MATERIAL AND METHOD

We used 15 male albino guinea-pigs. Animals were selected from the central bioterium of the Medical School, Ribeirao Preto, Sao Paulo University (FMRP-USP). Animals were cared for according to the guidelines in the Manual on the Care and Use of Laboratory Animals of the Institute of Laboratory Animal Resources.^13^ The study protocol also was designed according to the ethical principles of the Brazilian College of Animal Experimentation (COBEA).

The organophosphorus substance Tamaron (metamidophos), sold by Bayer CropScience Ltd, was used for intoxication studies on the guinea-pigs.

The animal study ethics committee (CETEA) of the FMRP-USP approved the study; the protocol no. was 100/2008.

The animals were selected in the central bioterium of the FMRP-USP; their individual weight ranged from 300 to 500g, and the Preyer reflex was present.

Next, the guinea-pigs underwent external otoscopy to visualize the external auditory meatus (EAM). Animals with signs of acute external otitis or otitis media, or that had difficult to remove earwax, or that presented changes in the EAM, or an excessively narrow auditory canal for the probes, were excluded from the study. According to the literature,^14^ otitis media increases the rigidity of bones, making the tympanic bulla difficult to open for fixation, and may facilitate damage to other structures, such as the cochlea, the vestibular system, and the ossicles.

The animals were housed in the bioterium of the experimental surgery laboratory of the Surgery Department, FMRP-USP.

The guinea-pigs were allocated to three groups:

GROUP 1: three animals - six cochleae, saccules and utricles; a single daily intraperitoneal dose of distilled water was administered with the same volume as that of the dose of the toxic agrochemical for the weight of the animal during seven days.

GROUP 2: six animals - twelve cochleae, saccules and utricles; a single daily 0.3 mg/kg/day intraperitoneal dose of the toxic agrochemical was administered during seven consecutive days.

GROUP 3: six animals - twelve cochleae, saccules and utricles; a single daily 3.0 mg/kg/day intraperitoneal dose of the toxic agrochemical was administered during seven consecutive days.

The guinea-pigs were weighed daily immediately before administering the drugs to correctly estimate the doses.

The recommended precaution measures in the insert were adopted when administering the daily doses to the guinea-pigs because of the importance of individual protection equipment in avoiding contact with agrochemicals.

On the following day after the last dose of the drug or distilled water were administered in each group, the guinea-pigs were anesthetized with intramuscular 2% xylazine chloridrate and 10% ketamine chloridrate and sacrificed by decapitation. The bullae were removed bilaterally and opened to expose vestibulocochlear structures.

The next step was to inject a 2.5% glutaraldehyde fixating solution through the round window of the cochlea. After this initial step, the material was washed five times with a 0.1M phosphate buffer solution and then microdissected to expose the cochlear spirae. The specimen was again immersed in a 0.1M phosphate buffer solution for 12 hours and washed with the same solution. The specimens were again fixated in a 1% osmium tetroxyde solution in a 0.1M phosphate buffer for 1 hour at 4°C. The material was then washed with 3 two to three baths of bidistilled water and then immersed in a 1% aqueous tannic acid solution during 1 hour at 4°C.

The specimens were dehydrated in successive 10-minute ethanol baths at increasing concentrations (50%, 70%, 90%, and 95%), followed by 3 20-minute baths with 100% ethanol; the specimens were left immersed in the last of these baths for 12 hours at room temperature.

Any remaining water in the samples was removed with a BAL-TEC - CPD 030 - Critical Point Dryer (Balzers, Liechtenstein) device, using the critical point process, in which the sample were placed in the chamber and covered with liquid carbon dioxide (CO_2_).

For adequate observation with a scanning electron microscope (SEM), the dissected and partially prepared structures were fixed in a metal cylindrical specimen-holder with conductive carbon paste to fix the cochleae in place, and with a carbon adhesive to fix the sacculae and utricles. The structures were then coated with a thin gold layer using a vaporization process with the BAL-TEC - SCD 050 - Sputter Coater (Balzers, Liechtenstein) device, to make them electrically conductive, as described in studies.^15^, ^16^, ^17^

The samples were then analyzed under a SEM -a JEOL Scanning Electron Microscope - JSM 5200. The structural analysis of cochleae was standardized, staring with the middle third of the first three cochlear spirae. The apical spira was not included in this study because of the natural disarray of OHC and inner hair cells (IHC), both in the “V” pattern and in cell queuing, especially in the second and third rows, making it difficult to study the presence and integrity of these cells.^14^^,^^17^

Cell integrity or injury was defined by analyzing their stereocilia. Cells with well-shaped and arranged stereociliae were considered normal. Cells with absent, deformed or disarranged stereociliar were considered as injured.^16^^,^^18^^,^^19^

## RESULTS

The OHC and IHC architecture and the saccules and utricles remained normal in group 1 guinea-pigs.

Scanning electron microscopy of the other two groups that were given agrochemicals revealed morphological changes in 83.3% of cochleae (group 2) and in 100% of cochleae (group 3); lesions were more extensive in the latter group. The rate of changes in the cilia, saccules and utricle in the vestibular labyrinth was 50% in group 2. Changes in the cilia and saccules reached 90% in the guinea-pigs; the change rate was 83% in the utricles of guinea-pigs in group 3.

The anatomical assessment of group 2 - given 0.3 mg/Kg/day - showed that the OHC were injured in the spira 3 (S3); injuries were ciliary distortion and disarray in a “V” pattern, and shortening or absence of the ciliae. Such changes were found in the 2nd and 3rd rows, particularly the latter. Changes in the IHC were seen in the basal spira, the spira 2 (S2) and S3; ciliae were present, but disarrayed.

**Figure 1:**
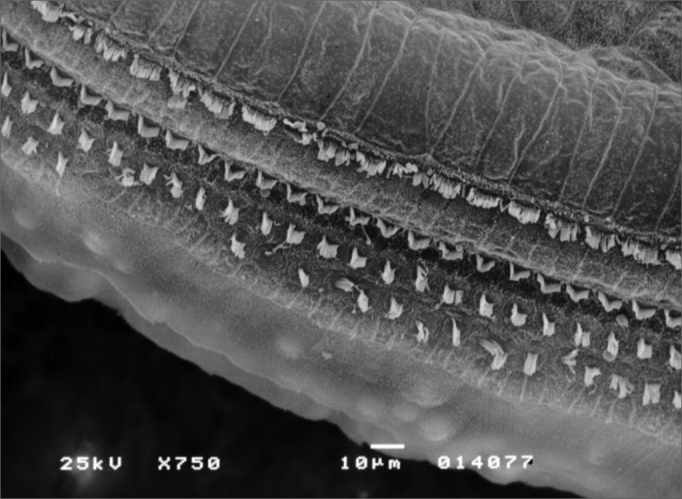
Photomicrography of the Corti organ in group 2 guinea-pigs, showing E3. 750 X magnification.

**Figure 2:**
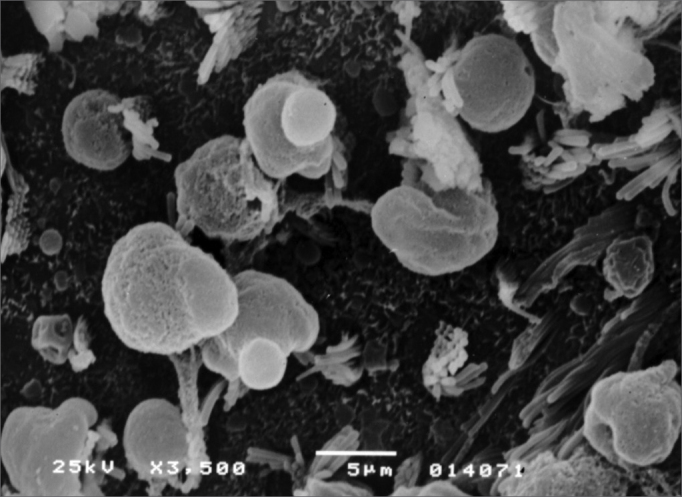
Photomicrography of the saccular macula in group 2 guinea-pigs. 3,500 X magnification.

**Figure 3:**
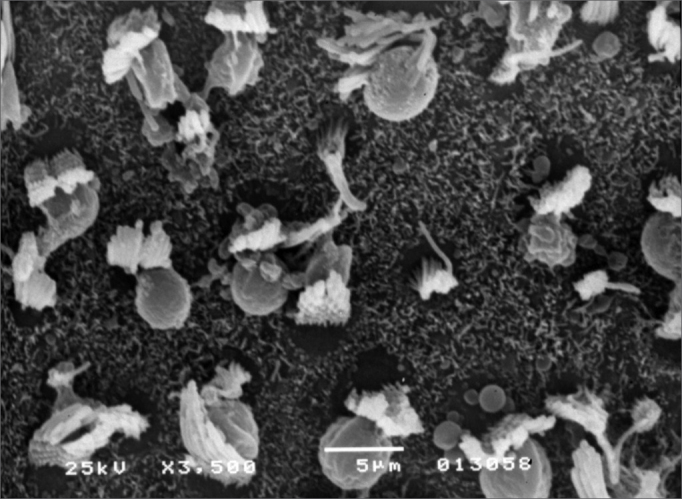
Photomicrography of the utricle macula in group 2 guinea-pigs. 3,500 X magnification.

Observed structural changes in the vestibule of the labyrinth of group 2 guinea-pigs were shortened and/or fused ciliae. Figure 1, Figure 2, Figure 3 show, in turn, the changes in the cochlea, saccules and utricle.

**Figure 4:**
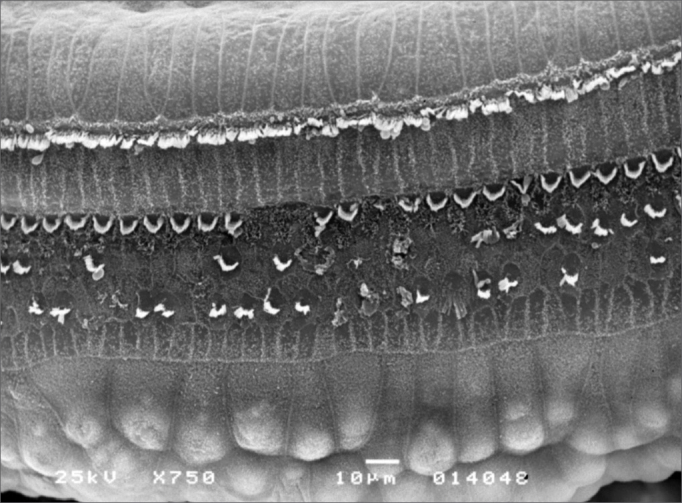
Photomicrography of the Corti organ in group 3 guinea-pigs, showing E3. 750 X magnification.

Morphological changes in group 3 - given 3.0 mg/Kg/day - were more extensive; OHC ciliae were absent or disarrayed in a “V” pattern, or folded, or one of the “V” arms was partially absent. Morphological changes in the basal spira involved only the 3rd row of OHC; in S2, the 2nd and 3rd row were more affected. In S3 all three OHC rows were severely affected. IHC ciliae also became irregular, shortened and fused in the three spira. Figure 4 shows the cochlear changes.

**Figure 5:**
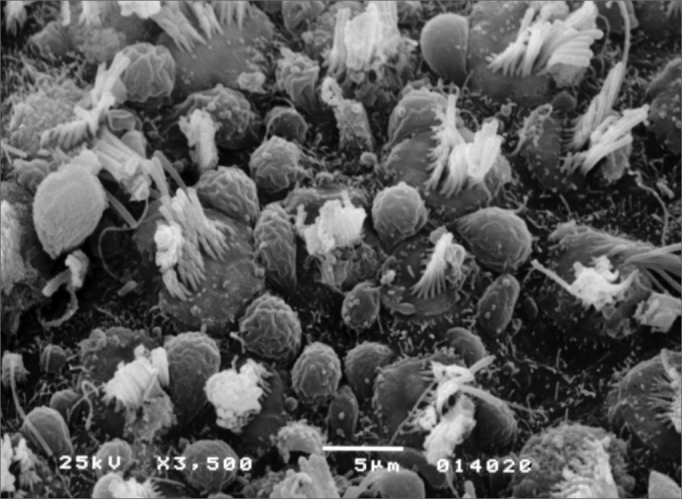
Photomicrography of the saccular macula in group 3 guinea-pigs. 3,500 X magnification.

**Figure 6:**
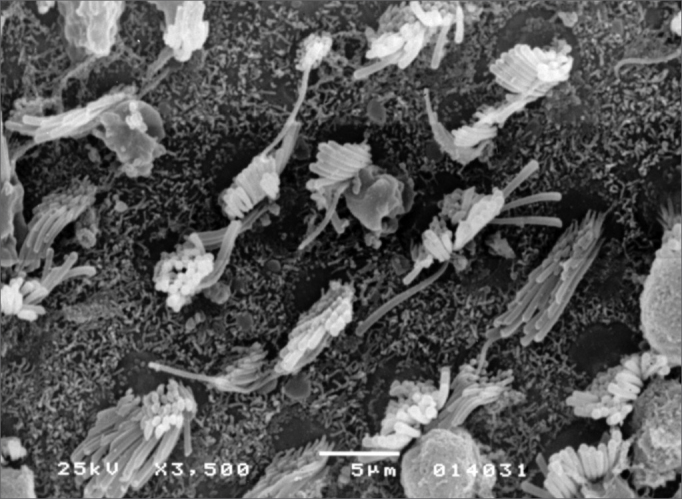
Photomicrography of the utricle macula in group 3 guinea-pigs. 3,500X magnification.

In the vestibular labyrinth, ciliary changes were shortened ciliae, ciliary fusion, and ciliary apoptosis. Figure 5, Figure 6 show the changes in the saccules and utricle.

## DISCUSSION

The adverse effects of agrochemical intoxication that we observed have been described in the literature. Studies on laboratory animals to analyze the effects of agrochemicals on the CNS have shown that chronic exposure to organophosphorus agents causes hypertrophy or the molecular layer of the cerebral cortex, and may cause neuron branches to taper off or be lost.^20^^,^^21^ Structural changes were observed in the cerebellum after a single acute intoxication episode; in this case, Purkinge cell apoptosis and structural damage to the cytoskeleton of surviving animals was observed.^22^ Another study of two organophosphorus insecticides given to female rats during pregnancy and lactation revealed that the development of teeth, fur and ears in newborn animals was altered, as were the postural, palmar and startle reflexes.^23^

Otological studies of animals are usually carried out on guinea-pigs and rats, because their ears are similar to human ears.^14^ We used guinea-pigs in our study because it is a docile and easily-handled animal with a reasonably sized middle and inner ear, which facilitates dissection and study; its peripheral auditory system is anatomically and pathologically similar to that of human beings.^15^^,^^17^^,^^18^ Another author^24^ has also suggested that guinea-pigs are used in studies of the labyrinth, because their vestibular system is markedly similar to that of humans.

We made a descriptive study of SEM findings, based on the method described in the literature by several authors^25^ that investigated the ototoxicity of drugs.

Based on SEM data, we inferred that the injury caused by an organophosphorus substance in the Corti organ affect mostly the OHC, and progress from the cochlear apex to its base. In the spirae we analyzed, the third row of OHC was always injured first, followed by the second and first rows. This injury sequence coincides with OCH height; S3 is injured first, followed by S2. The basal spira is third in number of changes. Cells in the apex were not studied, as cells in this spira are naturally disarrayed.

These findings confirm the tendency that lesions progress morphologically according to the degree of intoxication due to organophosphorus substances; there were more injuries in group 3 compared to group 2, as the former was given a higher dose of the agrochemical substance.

SEM observations in studies on guinea-pigs that were given ototoxic drugs diverge from those of our study. Injury predominated in the first two spirae (lower number of normal OHC, compared to S3) in amikacin exposure. Structural changes were more intense in the first OHC row, followed by the second row.^26^

These findings concur with those of other researchers,^18^ who showed that aminoglycosides cause injury to the Corti organ mostly in OHC, where the progression of injury is from the cochlear base to its apex; the first OHC row is injured first, followed by the second and third rows in turn.

Studies of cisplatin^25^ have shown extensive OHC injury in the basal cochlea, which were also found in another study,^19^ where the most evident changes were found in the basal spira (absence of ciliae in the first three rows of OHC), followed by S2 and S3 in turn. IHC ciliae were also injured; they were present, but disarrayed.

The morphological analysis of the injuries encountered in this study showed that the predominating changes were the absence and “V”-type deformities of stereociliae, which is similar to findings in other studies.^15^^,^^17^^,^^26^

The presence of the vestibulocochlear structural changes we found in the present study confirm that the organophosphorus agrochemical is toxic; this had previously been commented only in relation to altered function of the auditory and vestibular systems.

Several authors have described cases of sensorineural hearing loss due to exposure to agrochemical substances, ranging from mild to moderate loss.^27^^,^^28^ It has been reported that acute organophosphorus intoxication may cause profound bilateral hearing loss.^29^ The literature has reported that higher frequency auditory thresholds are more prone to damage;^1^^,^^11^^,^^30^ however, changes have also been noted at 1,000 and 2,000 Hz1.

Studies of rural workers have shown that the harmful effects of agrochemicals may involve the vestibular system. Some authors have noted the presence of altered bodily balance in the form of irritative peripheral vestibular syndrome.^11^ Other studies^31^, ^32^, ^33^ have also reported loss of postural stability, suggesting a possible subclinical effect on proprioception and the vestibular system.

Studies on the ototoxicity - cochleotoxicity and vestibulotoxicity - of agrochemicals add to our knowledge of the anatomy and physiology of the inner ear and the auditory pathways; this is also a first step to discover more efficient measure to avoid damage and to protect the Corti organ.

Our results underlie the need for additional research in animals from which data may be applicable gleaned for human beings to investigate in greater depth the effect of acute and chronic agrochemical intoxication on the body, especially on the auditory and vestibular systems.

## CONCLUSION

The observed morphological changes in the cochlea, saccules and utricle of guinea-pigs in the groups exposed to daily doses of an organophosphorus chemical shows that this agrochemical degrades the vestibulocochlear system.
